# Food Insecurity, telomere length and the potential modifying effects of social support in National Health and Nutrition Examination Survey

**DOI:** 10.1017/S1368980023002008

**Published:** 2023-12

**Authors:** Sarah M Lima, Xuefeng Ren, Lina Mu, Heather M Ochs-Balcom, Tia Palermo

**Affiliations:** Department of Epidemiology and Environmental Health, School of Public Health and Health Professions, State University of New York at Buffalo, Buffalo, NY 14214, USA

**Keywords:** Telomere length, Food insecurity, Social support, National Health and Nutrition Examination Survey

## Abstract

**Objective:**

Telomere length (TL) is a posited pathway through which chronic stress results in biological dysregulation and subsequent adverse health outcomes. Food insecurity is associated with shorter TL. Social support, which is defined by the size and function of an individual’s social network, is associated with better health outcomes. The present study assesses whether social support modifies the relationship between food security and TL.

**Design:**

Cross-sectional study design. Linear regression was used to assess the association between food insecurity and TL, stratified by social support level. A multiplicative interacted model was used to formally test modification.

**Setting:**

Data come from the National Health and Nutrition Examination Survey 1999–2000 and 2001–2002 waves.

**Participants:**

Adults aged 60 years and older who have measurements for TL.

**Results:**

Our sample comprised 2674 participants, and 63·5 % of the total sample had low social support, with 13·3 % being food insecure. In fully adjusted models, food insecurity was negatively though modestly associated (*P* = 0·13) with TL. Associations between food insecurity and TL were significantly modified by social support (interaction *P* = 0·026), whereby food insecurity had a stronger effect among individuals with high social support (coefficient = –0·099 (95 % CI: –0·161, –0·038)) compared to low social support (coefficient = –0·001, (95 % CI: –0·033, 0·032)).

**Conclusion:**

Food insecurity is modestly associated with shorter TL. Contrary to our hypothesis, food insecurity had more deleterious effects on TL among participants with high social support than low social support. Results may indicate that the food insecure population is a higher needs population, and increased social support reflects these needs rather than providing protective effects.

Telomeres are caps of nucleotide repeats at the ends of chromosomes that ensure genetic integrity during mitosis^(1,2)^. Telomeres naturally shorten through the lifespan as a result of cellular division and incomplete replication; thus, telomere length (TL) is a marker of biological age and ultimately long-term health^(3)^. Prior research has identified shorter TL to be associated with a range of health outcomes, including mortality, CVD, cancer progression, diabetes and obesity^(4–7)^. Along with ageing, exposure to external stress (physical and psychosocial stressors) can further accelerate telomere shortening through senescence-signalling pathways^(8)^. As such, TL is hypothesised to be a biomarker of chronic stress that causes downstream biological dysregulation and illness and thus may be a mediator of health outcomes^(2,8,9)^.

Socioeconomic status is associated with TL. Low socioeconomic status has been linked to shorter TL in adults, children and at the neighbourhood level^(10–12)^. Notably, in a recent prospective cohort study, parental socioeconomic status was positively associated with TL in newborns^(13)^. A systematic review of chronic social stress and TL found evidence that poverty-related stressors may induce telomere shortening^(14)^. It has been hypothesised chronic social stress leads to inflammation and oxidative stress and in turn causes DNA damage and telomere shortening^(9,15)^. Food insecurity, which is defined as an individual or household having an insufficient supply or access to safe and nutritious food needed for normal growth and to maintain a healthy life^(16)^, is linked to higher stress and is considered a chronic stressor^(17–19)^. In line with findings from other socioeconomic factors, food insecurity was recently found to be negatively associated with TL in a US representative sample^(20)^.

While chronic stress has adverse health consequences, evidence suggests social support is protective against chronic stress^(21)^. Social support is defined by two dimensions: (1) social structure, including network size and frequency of interactions, and (2) functional support, including emotional, financial and assistive support^(22)^. As social support mitigates effects of chronic stress^(23–25)^, it follows that increasing levels of social support may be protective of TL. In fact, loneliness and low social support was found to predict shorter TL^(26)^. Similar results were found at the neighbourhood level; individuals living in neighbourhoods with high social cohesion had longer TL than individuals in low social cohesion neighbourhoods^(27)^. Finally, another National Health and Nutrition Examination Survey (NHANES) study found that spousal support is related to TL, where unmarried participants had shorter TL after adjusting for other sources of social support, sociodemographic factors and comorbidities^(28)^.

Despite a growing body of literature indicating adverse effects of poverty on TL and protective effects from social support, there are no studies to our knowledge investigating whether social support mitigates effects of poverty or food insecurity on TL. In this paper, we assess social support as an effect modifier of food insecurity on TL (T/S ratio) using NHANES data. We hypothesise that high social support is positively associated with TL among food insecure individuals.

## Methods

### Data source

**Fig. 1:**
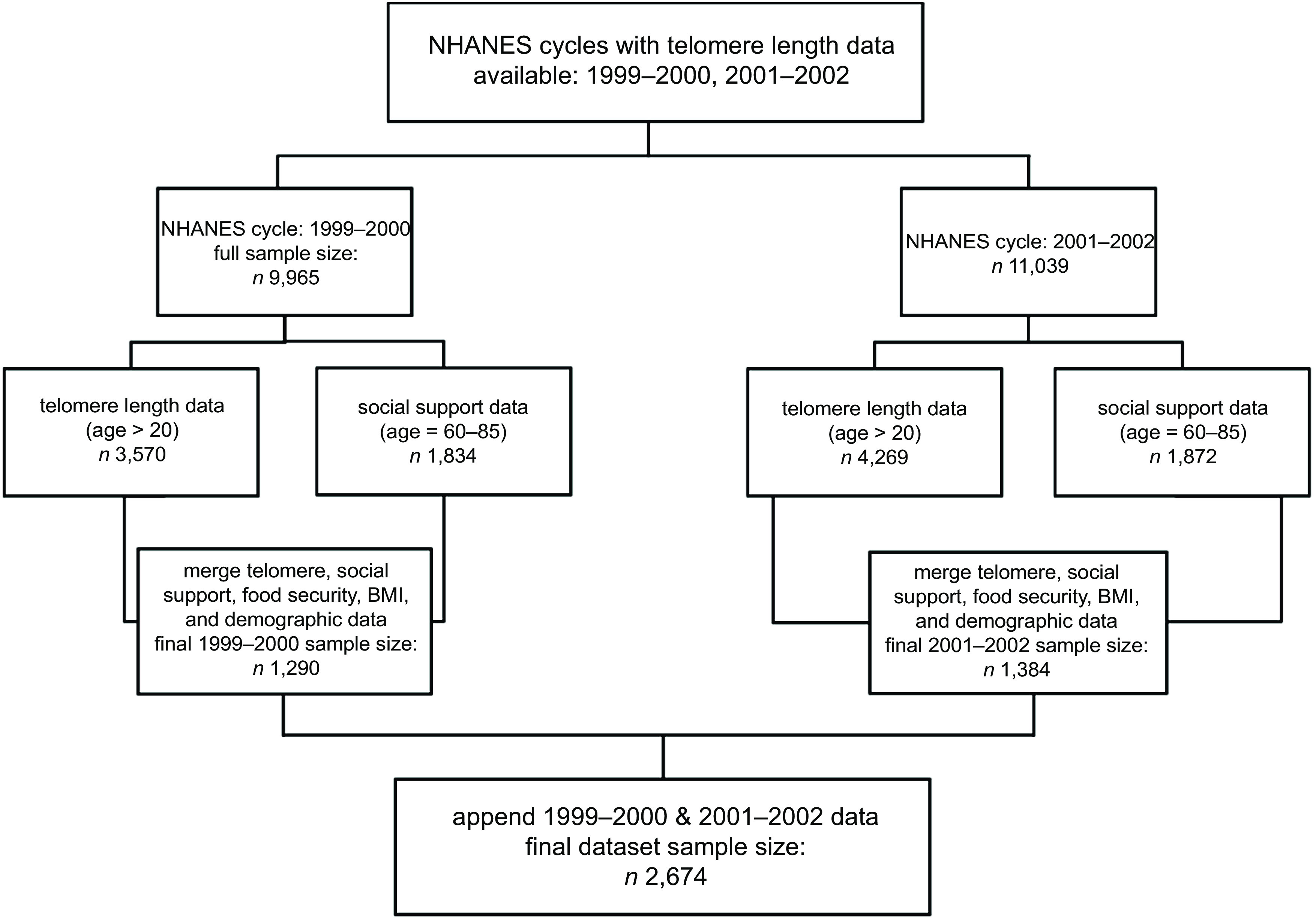
NHANES sample selection flow chart

Data come from the NHANES, which is conducted by the US National Center for Health Statistics^(29,30)^. The NHANES is designed to assess the health and nutritional status of the US population, combining interviews and physical examinations. NHANES is a cross-sectional survey that uses multistage probability sampling design for a nationally representative sample. Data are collected over two-year periods through survey interviews and examinations by medical personnel. We merged 1999–2002 NHANES data on TL (available among a sub-sample of respondents aged 20 years and older between 1999–2002), food security (available for full sample), social support (available for respondents aged 60 years and older) and participant demographics (available for all respondents). Our sub-sample for analysis corresponds to the years and age range with available data on all of these modules of interest (e.g. adults aged 60 and older in the 1999–2002 surveys). Figure 1 depicts the sample selection flow chart. Given data are de-identified and publicly available, this study was exempt from IRB review.

### Measures

Telomere assays have been described in detail previously^(31)^. Leucocyte TL measurements were obtained for two NHANES cycles using PCR method to measure TL relative to standard reference DNA (T/S ratio). We log-transformed the T/S ratio for a normally distributed TL variable.

One adult member per household was asked about food security over the past 12 months. Food security is categorised by NHANES as ‘food secure’, ‘marginally food secure’, ‘food insecure without hunger’, ‘food insecure with hunger’. For this study, we combined ‘food secure’ with ‘marginally food secure’, and ‘food insecure without hunger’ with ‘food insecure with hunger’ for a binary food security variable (secure *v*. insecure). We conducted sensitivity analyses to assess different combinations of food security.

NHANES social support questions were pulled from the Yale Health and Aging Study and Social Network Index – Alameda County Study^(29,30)^. Consistent with prior research on social support in NHANES^(28,32,33)^, we created a social support index comprised of four questions that were each assigned one point if answered in the affirmative: marital status (married, not married); can count on someone to provide emotional support (yes, no); has someone they can count on if they need some extra help financially (yes, no); and having more than five friends (yes, no). The social support index ranged from zero to four and was dichotomised, with a score of three or higher defined as high social support.

### Statistical analysis

We assessed means of demographic characteristics among the full sample and then stratified by food insecurity (food secure *v*. food insecure). To test whether demographic characteristics differed by food security status, we conducted bivariate analyses using *t* test and chi-square tests. A kernel density plot was used to assess the distribution of mean TL by social support. We used linear regression models to assess associations between food security, social support and log-transformed TL. In our base model, we regressed logged TL onto food insecurity, social support and covariates. Our covariates included age (continuous), female sex, self-reported race/ethnicity (non-Hispanic White (reference), non-Hispanic Black, Hispanic, other), educational attainment (range: < 9th grade to ≥ college graduate) and BMI (as calculated by NHANES using height and weight measurements). Race-ethnicity was intentionally included in statistical analyses as a potential confounder of the relationship between food insecurity and TL based on the following criteria: (1) the prevalence of food insecurity is 2–3 times greater in Hispanic and non-Hispanic Black households compared to non-Hispanic White households^(34)^, (2) average TL differs according race-ethnicity and is hypothesised to be a potential mechanism by which racial discrimination results in adverse health outcomes^(35)^, and (3) we do not have reason to believe race-ethnicity would be an intermediary of the causal pathway between food insecurity and TL.

To assess whether social support modifies the association between food insecurity and TL, we ran an interacted model using a cross-product term between food insecurity and social support. We then stratified our models by food insecurity to evaluate the direction and strength of modification by social support.

For sensitivity analyses, we ran our base model and interaction model stratified by median age (70 years) and ran a model adjusted for smoking status (categorised as never (reference), former, current). While NHANES is nationally representative, our sub-sample for analysis is not; it is limited by data availability, including observations with information on TL and social support (both are sub-samples of the overall dataset). Our goal in this analysis is not to describe nationally representative estimates, but rather to examine a relationship between characteristics. Thus, we do not use sampling weights in this analysis^(36)^. All analyses were performed using STATA SE 17.0.

## Results

**Fig. 2:**
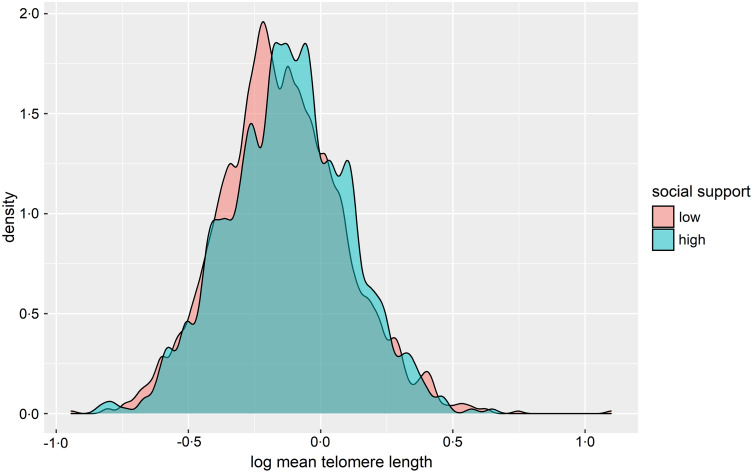
Distribution of telomere length by social support level (*P* = 0·07)

Our sample for analysis included 2674 participants (Table 1). The average age was 71·5 years, 49·1 % of the total sample was female, the majority was non-Hispanic White (58·3 %), and 63·5 % of the total sample was categorised as having low social support. Of the 2674 total participants, 356 (13·3 %) were food insecure. Food insecurity status differed with respect to age, race/ethnicity, educational attainment, BMI, income, marital status and social support (*P* < 0·05). TL did not significantly differ by food insecurity status; however, TL significantly differed according to social support level (*P* = 0·07), such that the high social support group had longer TL (Fig. 2).

**Table 1:** Demographic descriptive statistics

	Overall, *n* 2674			Food secure, *n* 2236			Food insecure, *n* 356			
	*n*	%	95 % CI	*n*	%	95 % CI	*n*	%	95 % CI	*P*-val
Telomere length			0·89, 0·91			0·89, 0·91			0·87, 0·91	
Mean	0·9			0·90			0·89			0·368
sd	0·22			0·22			0·21			
Age			71·2, 71·8			71·4, 72·1			69·0, 70·5	
Mean	71·5			71·7			69·7			< 0·001
sd	7·89			7·94			7·29			
< 70 years	1209	45·2 %		992	44·4 %		185	52·0 %		0·007
≥ 70 years	1465	54·8 %		1244	55·6 %		171	48·0 %		
Female	1312	49·1 %		1082	48·4 %		188	52·8 %		0·121
Race-ethnicity										< 0·001
Non-Hispanic White	1559	58·3 %		1440	64·4 %		84	23·6 %		
Non-Hispanic Black	397	14·9 %		324	14·5 %		59	16·6 %		
Hispanic	664	24·8 %		426	19·1 %		208	58·4 %		
Other	54	2·0 %		46	2·1 %		5	1·4 %		
Education										< 0·001
< 9th grade	690	25·9 %		459	20·6 %		205	57·6 %		
9–12th grade (no diploma)	466	17·5 %		386	17·3 %		64	18·0 %		
High school/GED	619	23·2 %		556	24·9 %		50	14·0 %		
Some college/assoc. degree	492	18·4 %		450	20·2 %		26	7·3 %		
College graduate & higher	401	15·0 %		381	17·1 %		11	3·1 %		
BMI			28·0, 28·5			27·9, 28·4			28·2, 29·4	
Mean	28·2			28·2			28·8			0·044
sd	5·37			5·40			5·27			
Income (in thousand dollars)			33·7, 35·6			35·8, 37·9			17·2, 20·3	
Mean	34·66			36·87			18·73			< 0·001
sd	23·57			23·84			13·99			
Married	1597	62·4 %		1362	63·8 %		189	54·8 %		0·001
Social support										
High support	808	30·2 %		720	34·4 %		61	18·2 %		< 0·001
Low support	1699	63·5 %		1373	65·6 %		274	81·8 %		

Quantitative variables by social support: independent *t* test. Qualitative variables by social support: chi-square.

In our base model among the full sample, food insecurity was not significantly associated with TL, adjusting for social support and covariates (Table 2). However, in our stratified model (Table 3), we found significant negative associations between food insecurity and TL among the high social support group (*β* = –0·099; 95 % CI = –0·16, –0·04), but a non-significant relationship among the low support group (*β* = –0·001; 95 % CI = –0·03, 0·03). Results from our interacted model showed significant modification by social support on the multiplicative scale (*β* = –0·076, *P* = 0·026; Table 3).

**Table 2:** Food insecurity, high social support and telomere length

Food insecurity and telomere length, adjusted for social support (*n* 2280)		
	*β*	95 % CI
Food insecure	−0·022	–0·051, 0·007
High social support	0·014	–0·006, 0·034
Age	−0·007	–0·008, –0·006
Female	0·053	0·034, 0·072
Non-Hispanic Black	0·024	–0·004, 0·052
Hispanic	−0·021	–0·047, 0·005
Other race	−0·017	–0·082, 0·049
BMI	0·000	–0·002, 0·001
Education	0·003	–0·004, 0·011

**Table 3:** Effect modification by social support of food insecurity on telomere length

	High social support *n* 313		Low social support *n* 1976	
	*β*	95 % CI	*β*	95 % CI
Food insecure	−0·099	–0·161, –0·038	−0·001	–0·033, 0·032
Age	−0·007	–0·01, –0·005	−0·007	–0·009, –0·006
Female	0·044	0·012, 0·076	0·056	0·033, 0·079
Non-Hispanic Black	0·058	–0·001, 0·118	0·012	–0·02, 0·045
Hispanic	0·026	–0·018, 0·071	−0·043	–0·075, –0·011
Other	−0·080	–0·179, 0·02	0·027	–0·059, 0·114
BMI	−0·003	–0·006, 0·00	0·00	–0·002, 0·003
Education	0·004	–0·008, 0·017	0·001	–0·008, 0·011

Cross product *food insecure* × *high social support*: coefficient = –0·076, *P* = 0·026.

Results from our sensitivity analysis, where we first stratified our base and interacted models by median age (70 years), were consistent with our main results in terms of direction of the coefficients, but the coefficients on the interactions were significant at the 5 % level only among those under 70 years of age (Appendices 1 & 2). Findings were robust to the inclusion of smoking status as a covariate (results not shown).

## Discussion

This is the first study to evaluate whether social support modifies the food insecurity–TL relationship. We used nationally representative data from US to investigate whether social support mitigates the negative association between food insecurity and TL. Food insecurity was significantly associated with shorter telomeres. However, contrary to our hypothesis, we found that food insecurity had a more deleterious association with TL among participants with high social support. Further, food insecurity was not associated with TL among participants with low social support.

In alignment with prior literature, we found an inverse relationship between food security and TL, though results were only significant among the sub-sample reporting high social support, and not among the full sample^(20,37)^. Our results are also consistent with prior literature indicating age and sex are associated with TL^(35,38)^. Chronic stress, which can result from discrimination, food insecurity and poverty-related factors, has commonly been shown to have adverse effects on TL, with some evidence that stress from poverty may induce attrition^(13–15,31)^. A recent study, also using NHANES, demonstrated food insecure individuals had significantly lower TL compared to food secure individuals^(20)^. However, in that study, this relationship was only significant among younger participants (ages 25–44 years), whereas our study found a significant relationship among those aged 60–85 years. Another mixed finding study conducted among Mesoamerican parents and children found increasing levels of food insecurity was significantly associated with shorter TL among fathers, but was not among mothers or children^(37)^. Further, maternal food insecurity was not found to be associated with newborn TL in a cohort of California mothers^(39)^. Together, these results indicate food insecurity may be differentially deleterious to TL across the lifespan.

Findings from the effect modification analyses are in contrast to our hypothesis. Similar to prior literature^(10,26,28)^, we found participants with high social support have longer TL compared to those with low social support. However, while we found evidence of a modifying effect from social support, the adverse effect of food insecurity was found only among the high social support group. This was an unexpected result given the large body of research indicating the protective effects of social support on health^(25,40–42)^. Studies on TL, in particular, have found such protection from social support^(28,43,44)^. Notably, the *Adults in the Making* trial, a family-based prevention programme focused on improving family social support and resilience, showed a significant difference in TL attrition over 5 years between trial arm, such that intervention participants did not experience a reduction in TL in contrast to controls^(45)^.

Given the evidence of protective effects from social support, along with the cross-sectional design of this study, our finding of more deleterious effects from food insecurity among individuals with high social support suggests social support could reflect endogeneity in the observed relationship. The food insecure population is generally a higher needs population, and increased social support may be reflective of increased needs rather than protective effects. Thus, longitudinal data, where changes in food security, TL and social support could be observed, would better identify the causal direction of this relationship.

### Limitations

Limitations of this study should be considered in interpreting the results. First, the study sample is comprised of older individuals (60–85 years old) due to NHANES asking the social support module to only this age group, which presents multiple limitations. Telomeres shorten with age^(3)^; thus, the older aged sample further obscures potential causal inferences on the relationship between food insecurity, TL and social support. Further, the older age may distort the level of social support compared to the participants’ average lifetime social support, particularly given widowing, which was scored as 0 in the creation of the social support index. Finally, the older age range results in lower generalisability and prevents any investigation across the life course. Given this array of limitations from the older sample, future research should include a more diverse age range to investigate the relationship between food insecurity and TL. Second, the social support measure used an index developed from four NHANES questions, which may not be as comprehensive as validated social support scales, such as the Multidimensional Scale of Perceived Social Support. However, a prior study similarly created a social support index using NHANES data and dichotomised the scale for analysis^(28,46)^. Third, data collection took place 20 years ago, as TL was only measured in those two NHANES cycles. Ideally, there would be more cycles with TL measures to improve sample size and power, particularly given the reduced sample size from NHANES’ age restriction of social support questions. Further, the prevalence of food insecurity and social support have changed over time^(47,48)^. However, we do not believe this would alter the relationship observed between food insecurity and social support. Relatedly, the US Department of Agriculture definition of food security changed from a binary to four-level categorical variable in 2006, but NHANES used this four-level variable in the 1999–2002 cycles and continues to do so^(49)^. Thus, the operationalisation of food security in the present study does not differ from current US Department of Agriculture standards.

### Conclusion

We found food insecurity was associated with shorter TL in a nationally representative sample from 1999–2001. There is evidence that social support modifies this relationship, such that food insecurity demonstrated a more adverse relationship with TL among the high social support group as compared to the low. While this finding is in contrast to our hypothesis, this finding suggests higher social support may be an indicator of higher needs and thus higher vulnerability rather than providing protection in this population. These results emphasise the consequences of food insecurity on health. Future research is needed to further examine the relationship between food insecurity and social support on TL, particularly in a younger, working-age sample and with longitudinal data to better identify the causal relationship. TL offers a direct biological connection between food insecurity and long-term health outcomes. As such, initiatives that increase food security, such as the social safety net, should be improved to prevent and abate adverse health outcomes and chronic diseases.

## Supplementary material

For supplementary material accompanying this paper, visit https://doi.org/10.1017/S1368980023002008
